# Effects of field releases of *Neoseiulus barkeri* on *Megalurothrips usitatus* abundance and arthropod diversity

**DOI:** 10.1038/s41598-024-64740-y

**Published:** 2024-06-20

**Authors:** YuanMing Chi, Chen Yu, MingYue Feng, Kai Shu, YiLin Zhu, WangPeng Shi

**Affiliations:** 1 Sanya Institute of China Agricultural University, Sanya, 572025 Hainan China; 2 Department of Entomology and MOA Key Lab of Pest Monitoring and Green Management, College of Plant Protection, China Agricultural University, Beijing, 100193 China

**Keywords:** Non-target arthropods, Biological control, Phytoseiidae, Functional response, Shannon’s diversity index, Agroecology, Behavioural ecology, Biodiversity, Ecology

## Abstract

*Megalurothrips usitatus* (Bagnall) (Thysanoptera: Thripidae) is an important pest in *Vigna unguiculata* (L.) Walp. *Neoseiulus barkeri* (Hughes) (Acari: Phytoseiidae) is widely used for control of pest mites and insects worldwide. We evaluated its effect on *M. usitatus* when predators (*N. barkeri*) or insecticides (Spinetoram) were applied in the fields. *Neoseiulus barkeri* Hughes consumed 80% of *M. usitatus* prey offered within 6 h, and predation showed Type III functional response with prey density. The maximum consumption of *N. barkeri* was 27.29 ± 1.02 individuals per d per arena (1.5 cm diameter), while the optimal prey density for the predatory mite was 10.35 ± 0.68 individuals per d per arena (1.5 cm diameter). The developmental duration of *N. barkeri* fed with *M. usitatus* was significantly shorter than those fed with the dried fruit mite, *Carpoglyphus lactis* (L.) (Acari: Astigmata). In field trials, the efficiency of *N. barkeri* against *M. usitatus* was not significantly different from that of applications of the insecticide spinetoram. Biodiversity of other insects in treated fields was assessed, and there were 21 insect species in garden plots treated with *N. barkeri* releases. The total abundance (N), Shannon’s diversity index (H), Pielou’s evenness index (J) and Simpson’s diversity index (D) of the garden plots treated with predatory mites were all significantly higher than that in the garden plots treated with spinetoram, where we found no species of predators or parasitoids and 7 herbivores. Our results show that *N. barkeri* is a potential means to control *M. usitatus* while preserving arthropod diversity at the level of treated gardens.

## Introduction

*Megalurothrips usitatus* (Bagnall), one of the most common flower-feeding thrips, known as the bean flower thrips, causes significant damage to many legumes and is the most important pest on cowpea in Southeast Asia, including Hainan Province in China^1–5^. Damage from high thrips densities includes premature shedding of flowers, causing significant cowpea quality decline and economic loss^6,7^. In 2020–2022, *Megalurothrips usitatus* caused losses of 240 million yuan per mu of land in Chengmai County, Hainan. Application of chemical pesticides is themain method used for control^8,9^; however, frequent applications have often selected for high resistance in *M. usitatus* and pose risks to non-target organisms^10–13^. Natural enemies of *M. usitatus* include *Beauveria bassiana* and *Orius sauteri* (Hemiptera: Anthocoridae)^1,5^.

Many phytoseiid mites are effective biological control agents that are widely used in agricultural production, especially greenhouses, based on their commercial production^14^. They are also an important part of natural control of phytophagous mites and insects in many crops^15^. Chinese predatory mites have been studied for approximately 50 years, and various species are now commercialized for application^16^. In recent years, the misuse and overuse of pesticides has resulted in resistance in many small piercing-sucking pests, such as mites, thrips, whiteflies, and aphids^17,18^. Also, frequent or large scale use of chemical pesticides is destructive to native natural enemies, potentially leading to pest outbreaks^19^. Releases of predatory mites can be effective in controlling some pests due to these predators’ small size, rapid development, strong reproductive ability, good predatory effect, and the similarity of their ecological niche with small sucking insect or mite pests^20,21^.

*Neoseiulus barkeri* is one of the predatory mites that are widely used to control pest mites and thrips in many countries^22–24^. This mite is the species reared in largest numbers in local production facilities, and *N. barkeri* has been used for pest control in many crops in China, where it is widely used^25,26^. In Inner Mongolia, it is used to control *Tetranychus truncatus* (Ehara)^27^. Wu et al.^28^ found that *N. barkeri* could be used with *Beauveria bassiana* against *Frankliniella occidentalis*^28^. *Tarsonemus confusus* Ewing is also preyed by *N. barkeri*^29^, but no field trials have been run to verify its use against this pest.

Non-target arthropods (NTAs) require protection from harmful pest management activities if their ecological services, such as regulation of arthropod populations, organic decomposition, organic material recycling, pollination, and biocontrol, are to be maintained^29–32^. When a new crop protection method is adopted, its possible side effects should be carefully considered^33^, particularly those on non-target organisms, such as parasitoids and predators, that play a key role in natural biological control. In this study, we evaluated the potential of *N. barkeri* to control *M. usitatus*. We also examined the impacts of such releases on biological diversity in cowpea fields.

## Material and methods

### Mite collection and rearing

Our *N. barkeri* and *M. usitatus* colonies, from which material used in these experiments was taken, originated in collections made on *V. unguiculata* in Sanya city, Hainan, China (N 18° 09′ 34″-L 108° 56′ 30″). *Neoseiulus barkeri* was subsequently cultured in plastic boxes (35 × 25 × 10 cm) that were filled halfway with water and contained a soaked sponge in the center. Filter paper and black plastic film were placed on top of the sponge. Mites were placed on the plastic film, where they were continuously reared by feeding them the dried fruit mite *Carpoglyphus lactis* (L.) (Acari: Astigmata) until stable populations developed. To maintain sufficient humidity within the rearing box and provide drinking water for the mites, the water level in the box was kept below the height of the sponge. The plastic boxes were kept in a climate-controlled chamber (PRX-1000B, BAIDIAN Equipment, Shanghai, China) maintained at a constant temperature of 25 °C ± 1 °C, 60% ± 5% relative humidity, and a photoperiod of 16L:8D (light: dark). *Megalurothrips usitatus* in our colony were fed on *V. unguiculata*, which also served as the oviposition substrate. The thrips colony was held at 26 ± 1 °C, 60% ± 5% relative humidity, and 16L:8D (light: dark) in a climate-controlled chamber.

### Experimental methods

#### Design of experimental arena

The experimental arena was modified from Munger cells^34^. In experiments on the attract success rate and the predation rate of adult females of *N. barkeri* on 1st instars of *M. usitatus*, the experimental cells consisted of a stack of three plates of acrylic plastic (5 length × 4 width × 0.3 height cm). The top plates had no holes, while the other two plates each had a circular hole (2.5 cm diameter) in the middle. In each such cell, one kidney bean leaf (4 length × 4 width cm) was placed on a filter paper (4 length × 5 width cm) and then positioned between the middle and bottom slide, forming a floor and sealing the enclosed cell. Two metal clips were used to hold the three plastic plates tightly together.

The cells used for the functional response test, and tests on the predation rates and nutritional benefits were the same as the cell described above, except that they were smaller (3.5 length × 2 width × 0.2 height cm). Trays containing the cells were held in a climate-controlled chamber at 25 °C ± 1 °C, 60% ± 5% relative humidity, and a 16L:8D photoperiod.

#### Exp. #1

The first of our three laboratory experiments evaluated the attack success and predation rate of adult females of *N. barkeri* on 1st instars of *M. usitatus*. The longevity of the predatory mite is 45–60 days, and from oviposition to adult emergence requires about 7 days. From 9 to14 days of age, they have maximal predatory efficiency*.* Gravid females of *N. barkeri* (9–14 days old) were transferred individually into modified Munger cells. A 1st instar *M. usitatus* (less than 7 h after egg hatch) was provided for each predator. Experiment #1 was run for 6 h, making observations of the prey every 5 min for the first 30 min and then every 20 min until the prey was eaten or the experiment was completed. Exp. #1 was repeated 15 times.

#### Exp. #2

This test assessed the functional response of *N. barkeri* adult females when offered 1st instars of *M. usitatus*. Newly mated females of *N. barkeri* (9–14-d old) were transferred individually into the modified Munger cells described above. The predatory mites were starved for 24 h to standardize their level of hunger before the experiment. Different densities of *M. usitatus* (5, 10, 15, 20, 25 and 30 1st instars, less than 7 h after hatching) were offered separately to *N. barkeri* adult females. Each treatment was replicated 10 times. After 24 h, the number of prey eaten was determined by counting the cadavers of fed-on 1st instar *M. usitatus*.

#### Exp. #3

In this test, we assessed the developmental duration of *N. barkeri* life stages reared on 1st instar *M. usitatus*. The dried fruit mite, *C. lactis*, was used as the control in our study of the developmental duration of *N. barkeri* on the pest thrips of interest because *C. lactis* is routinely used to rear predator mites. We collected eggs of *N. barkeri* and divided the *N. barkeri* larvae into 2 groups immediately after egg hatch. Each group was fed with one of the 2 prey species, *C. lactis* or 1st instar *M. usitatus*. After those *N. barkeri* mites became adults and mated, we collected the eggs laid by each test group separately. After those mite eggs hatched, larvae (20 for each group) were transferred as a treatment group into a cell, where the group was provided with sufficient food, either *C. lactis* or 1st instar *M. usitatus*, which were replenished every 12 h, at which times we observed the mites and recorded the durations of each life stage of each treatment group until all mites had died or molted to adults. Then, mites were held in a climate-controlled chamber at 25 ± 1 °C, 60% ± 5% relative humidity, and a 16L:8D photoperiod. Each treatment was replicated 20 times.

### Field trials

This trial was conducted using a completely randomized design, and it was carried out on land owned by the Sanya Institute of China Agricultural University, Sanya, Hainan, China, in 2022. From January 1, 2022 to April 4, 2022 in two cowpea fields of equal size (33 length × 20 width m). The temperature of the soil was a constant 25 °C ± 1 °C, the relative humidity was 60% ± 5%, and soil pH was 6.2–7.0. Both fields were surrounded with 2.5 m high, 80 mesh netting, and the soil was coverage with a silver plastic film. The cowpeas seeds were Nan Jiang Yi Hao, which were purchase form Sanya Academy of Tropical Agricultural Sciences.

Production practices applied in our plots followed local recommendations for cowpea production, as follows. We applied 3500–4500 kg of well-rotted organic fertilizer, 60–80 kg of calcium superphosphate, 30–40 kg of potassium sulfate, or 120–150 kg of wood ash per acre. Beds had two rows per bed, with a row spacing of 50–65 cm and holes spaced 20–25 cm apart. Each hole was sown with 4–5 seeds, which were covered with 2–3 cm of soil. Once the true leaves emerged, seedlings were inspected and gaps filled. Two or three robust seedlings were retained per hole. Irrigation was applied before installing supports. Organic fertilizer was applied to the furrows between cowpea rows Plants were irrigated as needed, especially subsequent to pod formation. During the growth period, plants were irrigated every 5–7 days. At the the 5–6 leaf stage, before stem elongation, supports for plant growth were added.

The fields for our experiments consisted of two contiguous with plantings buffer strip between the two treatment areas. When the cowpea seedlings were 50 cm tall, we started to release *N. barkeri* in one of the experimental fields, and used the other as a chemical control, treated with spinetoram. Both *N. barkeri* and spinetoram were applied at the same time when used for the first time, until the growing season was over. The *N. barkeri* mites were released every second week, and spinetoram was sprayed every week.

The determine impacts of treatment, 5 sampling points were randomly selected, and at each point, 3 small branches were selected twice a week. From each branch, 3 leaves are chosen, from the top, middle, and bottom parts, respectively, and after counting the total number of thrips at each sample point, the average value was calculated. Both species were stored in a wild-mouth sample bottles, with ethyl acetate, until examined in the laboratory.

To measure general insect diversity in these field plots, we used a sweep net (diameter is 40 cm, 50 sweeps per sample to collect insects from the foliage. Sweep netting was done twice a week until the end of the crop. Sweep net sampling was started after the flowering stage of cowpeas, and was done 5 times during the crop, generally at 3 days intervals. If it rained, the sweep netting and counting were omitted until the next scheduled sampling date.

### Statistical analysis

In Exp. 1 The attack success and predation rate were analysed by using Breslow tests, in SPSS 19.0, and the figure was made by Graphpad Prism 8.0.

In Exp. #2, the analysis of the functional response data was carried out in two phases. To determine type of the functional response curve, the proportion of prey consumed (N_e_/N_0_) was regressed against initial prey number using polynomial logistic regression (SAS 9.4) as per Eq. 1^35,36^.

1 $${{N_{e} } \mathord{\left/ {\vphantom {{N_{e} } {N_{0} }}} \right. \kern-0pt} {N_{0} }} = a + bN_{0} + cN_{0} + dN_{0} + e$$

where N_e_ = number of prey consumed; N_0_ = initial number of prey; a = intercept; b = linear; c = quadratic, and d = cubic coefficients.

Then, the functional response parameters were calculated using Eq. 2.

2 $${\text{Na }} = {\text{ ae}}^{{ - {\text{b}}/{\text{N}}}}$$

where Na = number of prey consumed; N = initial number of prey; a = maximum prey consumption; b = optimal search density^37^.

In Exp. #3, the developmental duration was compared between the two kinds of prey fed to the mite using t-tests for independent samples.

In the field trial, numbers of the pest *M. usitatus* in the two treatments were compared using t-tests for independent samples in SPSS 19.0. For insect diversity counts, before the analysis, numbers of different species were log(x) transformed. Three diversity indices—Shannon’s diversity index (H), Pielou’s evenness index (J), and Simpson’s diversity index (D)—were used to examine community structures between treatments. The Shannon Diversity Index is used to summarize the diversity of a population in which each member belongs to a unique group^38,39^. Pielou’s evenness index measures the evenness of species diversity or the equilibrium status of the species^40^. The value of the evenness index is generally between 0 and 1, and it can be used to represent the uniformity of the species distribution. When the evenness is low, that is, when the value is relatively small, it indicates that the distribution of species in the system is uneven. Conversely, when the value is relatively large, it indicates that the distribution of the species is even. Simpson's Diversity Index is a measure of diversity^39^. In ecology, it is often used to quantify the biodiversity in a habitat. It accounts for both the number of species present and the abundance of each species^39^. Simpson’s Diversity Index is often used to evaluate effects of agricultural treatments on non-target organisms^41,42^. All three indices are sensitive to the abundance of the most common or dominant species in the community. Linear mixed models were applied to analyse H, J, and D, using the lmer function of the R package lme4. On each sample date, mean values of N, H, J and D were compared using a one-way ANOVA to detect significant differences between fields treated with *N. barkeri* or spinetoram. The proportion of herbivores, predators, and parasitoids in each treatment is defined by the equation Pi = Ni/N, where Ni is the abundance of herbivores, predators, or parasitoids and N is the overall total abundance in each treatment. The figures were made by Graphpad Prism 8.0.

### Consent for participate

Agree

## Results

### Exp. #1. Attack success and predation rate

**Figure 1:**
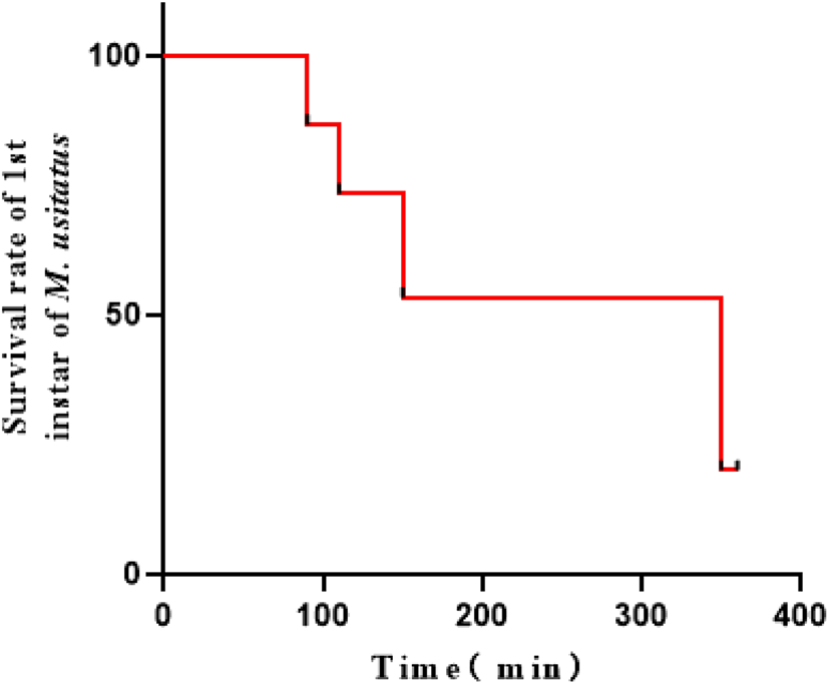
The survival of 1st instar of *Megalurothrips usitatus* when attacked by *Neoseiulus barkeri* females in a laboratory arena.

When 1st instar *M. usitatus* thrips were offered to female mites, 80% of *N. barkeri* from 12 groups preyed on *M. usitatus* within 6 h of observation (Breslow tests. *n* = 15, *p* = 0.03) (Fig. 1).

### Exp. #2. Functional response

**Figure 2:**
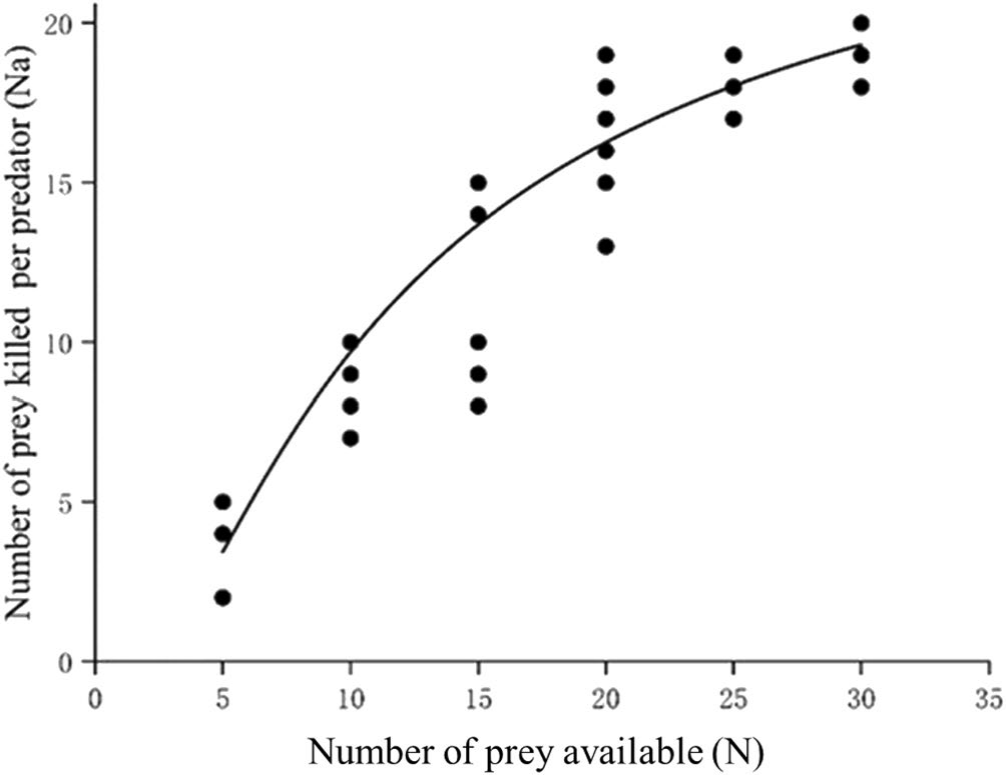
Functional response of *Neoseiulus barkeri* feeding on 1st instars of *Megalurothrips usitatus*.

In the functional response test, when the number of 1st instar *M. usitatus* thrips offered as prey increased from 5 to 30 per arena, the number of prey consumed per female *N. barkeri* increased from 4.30 to 19.10 (Table 1). At low prey density (below 15), female *N. barkeri* mites consumed most of the 1st instar thrips offered. The functional response curve plateaued at about 20 prey (Fig. 2) and then deaccelerated, indicating a Type III functional response (P1 > 0, P2 < 0) (Table 2). The maximum prey consumption rate was 27.29 ± 1.02 (individuals per·d per arena), and the optimal search density for each arena in this experiment was 10.35 ± 0.68 (individuals per·d per arena) (Table 3).

**Table 1:** Consumption of first instar of *Megalurothrips usitatus* by *Neoseiulus barkeri* at different prey densities (5, 10, 15, 20, 25 and 30).

Prey density	Consumption
5	4.30 ± 0.30e
10	9.40 ± 0.34d
15	12.50 ± 0.97c
20	16.50 ± 0.66b
25	18.60 ± 0.22a
30	19.10 ± 0.23a

Data are means ± SE. Means followed by different lower-case letters within a column are significantly different (one-way ANOVA).

**Table 2:** Estimates of coefficients in the logistic regression of the proportion killed of first instar of *Megalurothrips usitatus* by *Neoseiulus barkeri* as a function of initial prey density.

Type	Parameters	Estimate	SE	*χ2*	*p* value
III	Intercept (P0)	1.7081	1.2033	2.02	0.1557
	Linear (P1)	0.1004	0.2299	0.19	0.6623
	Quadratic(P2)	-0.0067	0.0131	0.26	0.6081
	Cubic (P3)	0.000071	0.000227	0.10	0.7565

**Table 3:** Functional response parameters of *Neoseiulus barkeri* feeding on the 1st instars of *Megalurothrips usitatus*.

Holling-III		
Maximum preyconsumption (individual per d per arena)	Optimal search density (individual per d per arena)	R^2^
27.29 ± 1.02	10.35 ± 0.68	0.90

Data are mean ± SE.

### Exp. #3. Life stage developmental times of predator on two prey species

When *N. barkeri* was reared on *M. usitatus* or *C. lactis*, there was no significant difference in the developmental time of the egg stage, as expected (t-test: *t* = − 0.171, *p* = 0.863) (Table 4). For the mite’s immature stages, larval stage duration was affected by prey species (t-test: *t* = 3.698, *p* = 0.001) (Table 4). The same was true for the protonymphs (t-test: *t* = 14.332, *p* < 0.001) (Table 4) and deutonymphs (t-test: *t* = 6.807, *p* < 0.001) (Table 4). Also, the total immature developmental duration of *N. barkeri* was significantly affected by prey species (t-test: *t* = 13.082, *p* < 0.001) (Table 4). When fed with 1st instar of *M. usitatus*, *N. barkeri* had shorter developmental duration (5.58 ± 0.12 d), with 90% survival. For the alternative prey, *C. lactis*, developmental duration was longer (8.95 ± 0.23 d), but survival (95%) was higher.

**Table 4:** Developmental times of *Neoseiulus barkeri* life stages fed on 1st instars of *Megalurothrips usitatus* or *Carpoglyphus lactis*. (in days, mean ± SE).

Diet	Developmental time (day)					
	Egg	Larvae	Protonymph	Deutonymph	Egg-to-adult	Survival%
*C. lactis*	1.92 ± 0.13^ns^	1.08 ± 0.10**	3.18 ± 0.10***	2.76 ± 0.16***	8.95 ± 0.23***	90
*M. usitatus*	1.94 ± 0.04	0.67 ± 0.06	1.50 ± 0.06	1.47 ± 0.10	5.58 ± 0.12	95

***: *p* < 0.001; **: *p* < 0.01; ns: *r* > 0.05.

### Field trials

**Figure 3:**
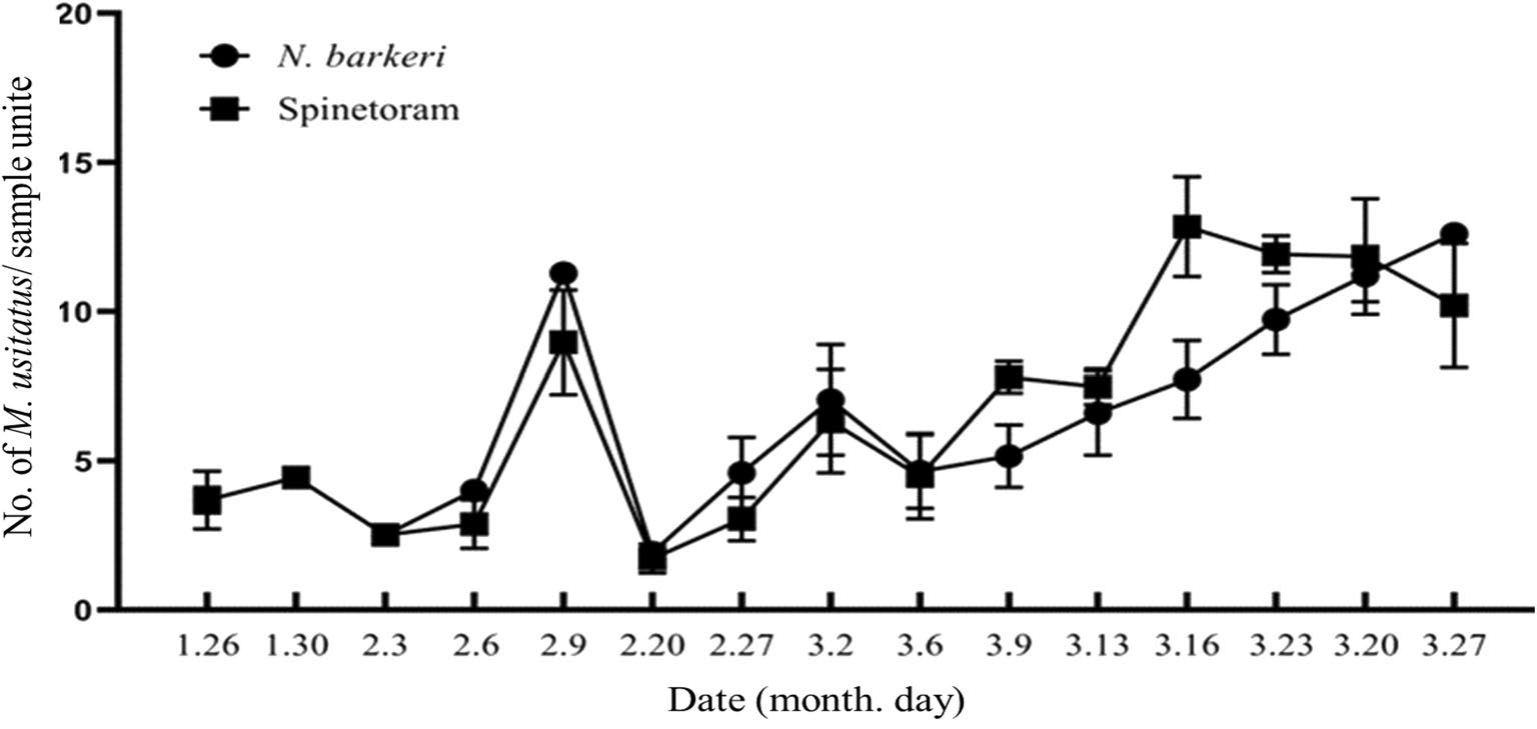
Densities of the pest thrips *Megalurothrips usitatus* in a cowpea field treated either with *Neoseiulus barkeri* releases (circles) or applications of spinetoram (squares).

The numbers of *M. usitatus* in samples from the two treatments (mites vs. spinetoram application) were not significantly different during the crop period (*T*-test: *t* = 1.760, *df* = 168, *p* = 0.08) (Fig. 3).

**Figure 4:**
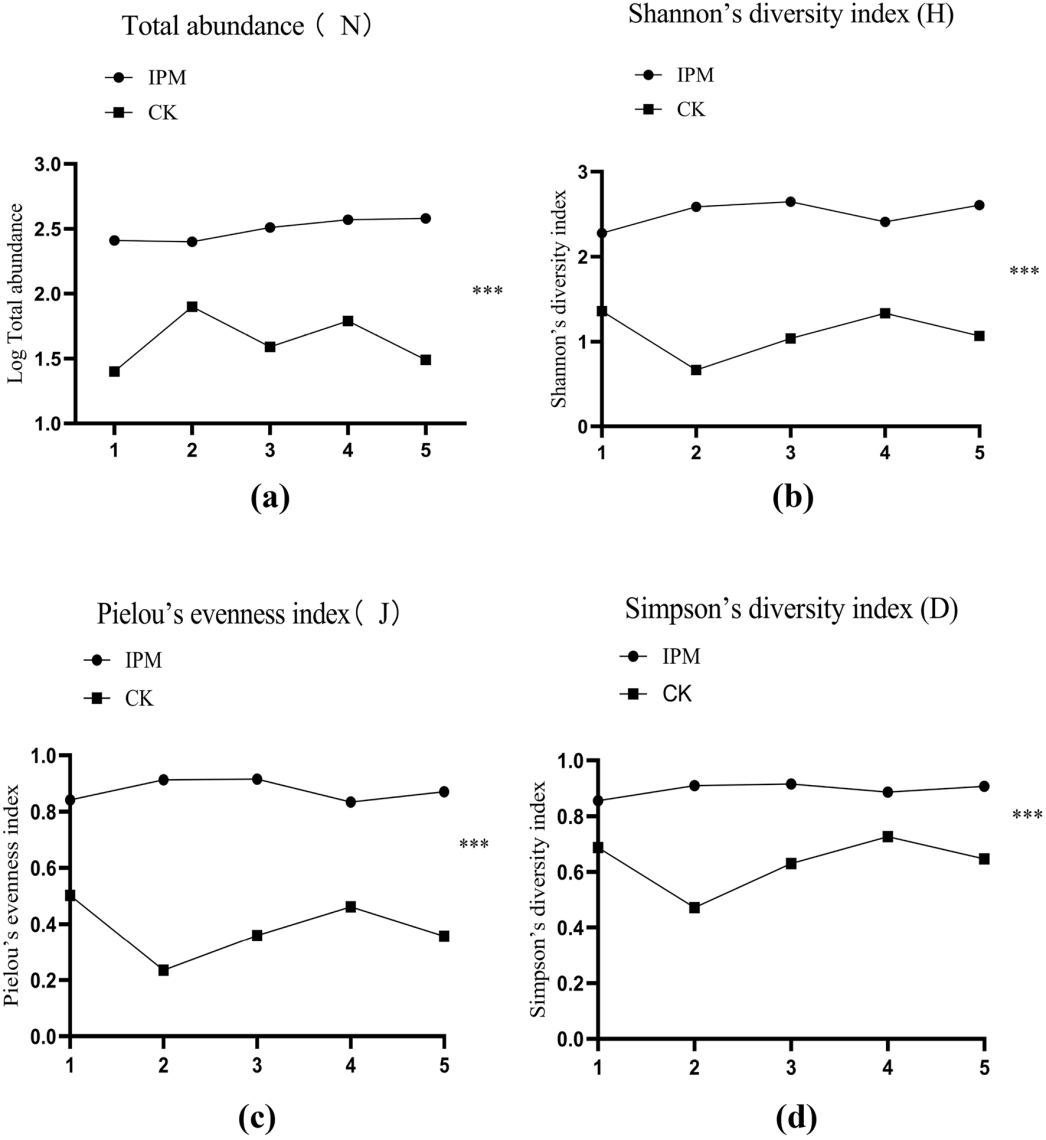
Changes in mean ± SE (n = 53) values of descriptors of the NTAs communities in two fields. (**a**) Total abundance; (**b**) Shannon’s diversity index; (**c**) Pielou’s evenness index; (**d**) Simpson’s diversity index. Statistically significant difference according to one-way ANOVA: ***p < 0.001.

In terms of impacton insect diversity (Table 5), total abundance (N) of non-target species was significantly higher in the mite-release field, than in the spinetoram-treated field (one-way ANOVA: LSD: *F*_1,8_ = 85.043, *p* < 0.001) (Fig. 4a). Also, Shannon’s diversity index (H) was significantly higher in the mite-release field (one-way ANOVA: LSD: *F*_1,8_ = 96.62, *p* < 0.001) (Fig. 4b), showing that the number of species present and the abundance of each species was higher in the absence of pesticide use. Similarly, Pielou’s evenness index in the *N. barkeri* field was closer to 1 (0.87 ± 0.02) in the spinetoram field (0.35 ± 0.05), and the treatments were significantly different (Fig. 4c) (one-way ANOVA: *F*_1,8_ = 97.70, *p* < 0.001). Finally, Simpson's diversity index (D) was signficantly higher in the mite-release field (Fig. 4d) (one-way ANOVA: *F*_1,8_ = 34.17, *p* < 0.001), the showing that the species distribution in the mite-release field was more even than in the spinetoram-treated field.

**Table 5:** The species list in *Neoseiulus barkeri* and spinetoram plots during the whole study period.

Species name	Ecological Group	Mite-release field	Spinetorm-treated field
*Trialeurodes vaporariorum*	H	**√**	**√**
*Systasis longula*	Par	**√**	
*Spodoptera litura*	H	**√**	
*Sogatella furcifera*	H	**√**	
*Rhyparochromus albomaculatus*	H	**√**	
*Nitidulidae latreille*	H	**√**	**√**
*Metochu abbreviatus*	H	**√**	
*Megalurothrips usitatus*	H	**√**	**√**
*Liriomyza trifolii*	H	**√**	**√**
*Harnischia longispuria*	H	**√**	
*Harmonia axyridis*	Pred	**√**	
*Frankliniella intonsa*	H	**√**	**√**
*Epuraea picinus*	H	**√**	
*Episyrphus balteatus*	Pred	**√**	
*Cyrtogaster clavicornis*	Par	**√**	
*Cotton leafhopper*	H	**√**	**√**
*Chrysomya megacephala*	H	**√**	
*Baeoentedon balios*	Par	**√**	
*Bactrocera hainanus*	H	**√**	
*Aphis craccivora*	H	**√**	**√**
*Aedes albopictus*	H	**√**

## Discussion

This study examined the potential of *N. barkeri* as a new predator for augmentative biocontrol of *M. usitatus* in chickpea fields. As a first step, as per Schausberger and Croft (2000), we confirmed the predator’s attraction to the prey and its predatory ability (attack rate) against the target prey^43^. In our experiment, *N. barkeri’s* attack rate on prey was high (80%). As the second step, we measured the functional response of the predator when offered the pest of interest^44^. In our study, *N. barkeri* showed a Type III functional response when offered first instars of *M. usitatus*. However, the types of functional responses of predatory mites towards their target prey and exact parameter values are not constant and can vary under different conditions. Li et al.^29^ found that when different life stages of *Tarsonemus confusus* (Ewing) (Acari: Tarsonemidae) were offered to *N. barkeri*, the predator showed different kinds of functional responses^29^. When adult females of *T. confusus* were offered, *N. barkeri* displayed a Type III functional response. The type III functional response showed by *N. barkeri* to our target prey suggests potential to regulate the pest’s populations^45,46^.

In our third experiment, we looked at the nutritional value of the target prey to support the development of *N. barkeri*. Effects of prey species on predator developmental duration are common in predatory mites and insects^47^. In fact, *C. lactis* can serve as food for many predator mites, including *Amblyseius andersoni* (Chant) (Acari: Phytoseiidae) and *Neoseiulus neoreticuloides* (Liang et Hu) (Acari: Phytoseiidae)^15,48^. We found that *N. barkeri* could complete its growth and development on both *C. lactis* and *M. usitatus*, but it did so more rapidly when fed first instars of *M. usitatus*.

For pest suppression in crop fields, biocontrol has an advantage over pesticide use in that it typically preserved higher insect and mite diversity in treated areas, which was the case here. The Shannon's diversity index, Pielou's evenness index, and Simpson's diversity index all showed higher NTOs density, richness, or evenness in the biocontrol chickpea field. All these indices are, however, sensitive to the abundance of the most common and dominant species in a community^37,49^. In our study, all four indicators of NTOs (N, H, J and D) were significantly different between treatments, but the number of the pest, *M. usitatus,* was not significantly different between treatments (Fig. 3), indicating that mite releases achieved control equivalent to the pesticide applications.

The biological control function provided by predators and parasitoids is the foundation of natural regulation of insect populations in agricultural ecosystems^50,51^. In our experiment, several predators and parasitoids were observed in the field where we released *N. barkeri*, including *Harmonia axyridis* (Pallas) (Coleoptera: Coccinellidae) and *Episyrphus balteatus* (de Geer) (Diptera: Syrphidae). In contrast, no predators or parasitoids were found in the field where spinetoram was applied regularly. While spinetoram is considered a low-risk product to mammals^7,52^, it harms many kinds of beneficial insects. The absence of these groups in the spinetoram-treated plot may have been due to either direct toxicity or the induction of avoidance^11^. Tang et al. (2021^53^) found that bumblebees exposed to even low concentrations of spinetoram showed effect on their physiology and gut microbes.

In conclusion, we found *N. barkeri* to be an effective and safe predator for control *M. usitatu*s. However, in pest control, relying solely on a single method is often insufficient for controlling all pests. Therefore, it is necessary to control thrips using *N. barkeri* in conjunction with other measures, such as *Beauveria bassiana* (Bals.-Criv.) Vuill and *Orius sauteri* Poppius^1,5^ or selective pesticides. Chemical pesticides should be reserved as an emergency measure, as sustainable pest management is best achieved through Integrated Pest Management programs based primarily on biological control.

## Data Availability

The data that support the findings of this study are available from the first author upon reasonable request.
